# Levodopa-Induced Changes in Electromyographic Patterns in Patients with Advanced Parkinson’s Disease

**DOI:** 10.3389/fneur.2018.00035

**Published:** 2018-02-05

**Authors:** Verneri Ruonala, Eero Pekkonen, Olavi Airaksinen, Markku Kankaanpää, Pasi A Karjalainen, Saara M Rissanen

**Affiliations:** ^1^Department of Applied Physics, University of Eastern Finland, Kuopio, Finland; ^2^Department of Clinical Neurosciences, Neurology, University of Helsinki, Helsinki University Hospital, Helsinki, Finland; ^3^Department of Physical Medicine and Rehabilitaton, Kuopio University Hospital, Kuopio, Finland; ^4^Department of Physical Medicine and Rehabilitaton, Tampere University Hospital, Tampere, Finland

**Keywords:** Parkinson’s disease, levodopa challenge test, medication, EMG, kinematic, wearable, PCA

## Abstract

Levodopa medication is the most efficient treatment for motor symptoms of Parkinson’s disease (PD). Levodopa significantly alleviates rigidity, rest tremor, and bradykinesia in PD. The severity of motor symptoms can be graded with UPDRS-III scale. Levodopa challenge test is routinely used to assess patients’ eligibility to deep-brain stimulation (DBS) in PD. Feasible and objective measurements to assess motor symptoms of PD during levodopa challenge test would be helpful in unifying the treatment. Twelve patients with advanced PD who were candidates for DBS treatment were recruited to the study. Measurements were done in four phases before and after levodopa challenge test. Rest tremor and rigidity were evaluated using UPDRS-III score. Electromyographic (EMG) signals from biceps brachii and kinematic signals from forearm were recorded with wireless measurement setup. The patients performed two different tasks: arm isometric tension and arm passive flexion–extension. The electromyographic and the kinematic signals were analyzed with parametric, principal component, and spectrum-based approaches. The principal component approach for isometric tension EMG signals showed significant decline in characteristics related to PD during levodopa challenge test. The spectral approach on passive flexion–extension EMG signals showed a significant decrease on involuntary muscle activity during the levodopa challenge test. Both effects were stronger during the levodopa challenge test compared to that of patients’ personal medication. There were no significant changes in the parametric approach for EMG and kinematic signals during the measurement. The results show that a wireless and wearable measurement and analysis can be used to study the effect of levodopa medication in advanced Parkinson’s disease.

## 1 Introduction

Parkinson’s disease (PD) is a progressive neurodegenerative disease mainly among the old with increasing incidence with age (1, 2). There is no cure for PD. The main symptoms of PD are rigidity, rest tremor, bradykinesia, and postural instability (3). Majority of patients with PD experience rest tremor during their course of disease. Rest tremor can be present either in the beginning or a latter phase of the disease, or the whole time. Rigidity is characterized by increased resistance in the limbs. Along with rest tremor, rigidity hinders activity of daily living (ADL) in PD. With appropriate treatment, it is possible to relieve the symptoms of the disease and thus improve ADL of PD patients to maintain their active life several years longer.

The symptoms of PD can be alleviated with multiple types of medication. Levodopa medication is currently the most efficient treatment for PD, and it alleviates the motor symptoms. COMT-inhibitors are often used to enhance the duration of levodopa treatment effect. Mild symptoms of PD can be treated with a combination of other medication, such as dopamine agonists and MAO-B inhibitors, alone or in combination (4). This allows delaying of levodopa treatment, because duration and dose of levodopa treatment are associated with appearance of dyskinesia and motor fluctuations. Up to 50% of PD patients experience dyskinesia or motor fluctuations within 5 years from the onset of levodopa medication (4).

When motor symptoms can no longer be adequately controlled with medication, deep-brain stimulation (DBS) can be introduced. Electrodes are implanted in either subthalamic nucleus (STN) or internal segment of globus pallidus (5, 6) for dyskinesia and motor symptoms or to ventral intermediate thalamic nucleus (VIM) (7) for tremor control, to give continuous electrical stimulation via stimulation device. DBS has been shown to be more efficient than optimal medication to control motor fluctuations and dyskinesias, when the patient selection is done correctly (8). Levodopa challenge test is the most important single test to assess efficacy of DBS in advanced PD. Positive levodopa challenge test predicts successful outcome from DBS treatment (5, 9).

The symptoms of PD can be assessed by using Unified Parkinson’s Disease Rating Scale (UPDRS). UPDRS is a well-established rating scale to assess the multitude of PD symptoms. The third part, UPDRS-III, is based on motor assessment (0–108 points). Levodopa challenge test can be used to determine the effect of levodopa in patients with PD. The UPDRS-III score is determined before the dosage and approximately 30–60 min after the dosage of levodopa, when the medication effect is maximal. Over 30% decrease of UPDRS-III score in challenge test is generally regarded positive to introduce DBS in PD (5, 9).

Surface electromyographic (EMG) and kinematic methods have been established during last two decades for the clinical research of PD, and they can extract multitude features (10). It has been shown that the EMG signals of PD patients have different characteristics compared to healthy controls. The complexity of signals is reduced (11, 12), and more rhythmic bursts and pattern like behavior has been observed (13). Kinematic measurements are sensitive for tremor patterns. EMG and kinematic based analyses have been used to observe gait (14), REM sleep (15), medication response (13, 16–18), and DBS treatment (17, 19, 20) in PD. During recent years, the measurement devices have become smaller, portable, and wireless. This has made the measurements more feasible, thus longer and more measurements are available. Methods to classify parkinsonian symptoms during unconstrained activity have been presented (21–23). EMG and kinematic methods have been used to recognize levodopa-induced dyskinesias during medication response (24–26).

Traditional methods for analyzing the EMG and the kinematic signals include amplitude and spectral-based measures. These methods allow for the determination of the strength of muscle activation, muscle conduction velocity, firing rate of motor units, and fatigue. The kinematic signals can be quantified with amplitude and power measures. There are newer techniques for analyzing the EMG and the kinematic signals that include linear and non-linear parametrizations as well as methods which are statistics related. These methods focus more on the morphology of the EMG signal than amplitude and frequency. The EMG and the kinematic signals in PD have been studied with amplitude and spectral based methods (15, 17, 27–31), wavelet-based approaches (32–34), linear and non-linear parameters (11, 20), EMG-burst shape analysis (13, 35, 36) and principal component approach (37, 38).

In this study, we measure and analyze EMG and kinematic signals during isometric arm tension task with linear and non-linear methods as they have been proven to be effective for analyzing signals from patients with PD (12). For analyzing EMG signals during passive flexion–extension of arm, we use spectrum-based methods since they are well established and robust enough to analyze non-stationary signals during arm movement. There are two purposes for the present study: to devise a method that consists of measurements and analysis to objectively assess the levodopa challenge test and to prove that a wearable and wireless measurement can be used for monitoring the treatment of PD.

## 2 Materials and Methods

### 2.1 Subjects

After a written informed consent, EMG and kinematic signals of 12 (8 males, 4 females) patients with advanced PD were measured (Table 1). The UPDRS-III score was determined during the measurement to estimate the benefit of DBS for the patient. The patients had DBS later if they met all the selection criteria. The study was approved by human ethics committee of the Kuopio University Hospital. The age of the patients was (58 ± 7) (mean ± SD) years and they had had the PD diagnosis (9 ± 3) years before the measurement. The UPDRS-III score for the patients was (37 ± 8) before the administration and (13 ± 5) after 60 min of administration of levodopa.

**Table 1:** Patient demographic and medication data.

#	Sex	Age	Dur.	H and Y	UPDRS-III	UPDRS-III	LEDD	Test
				Med off	Med off	Med on		Dose
1	F	48	11	3	25	3	1,250	150
2	M	46	5	2	33	11	700	150
3	F	47	14	3	0	0	1,600	150
4	F	63	8	3	37	14	935	150
5	M	56	9	3	43	16	1,175	300
6	M	60	9	3	33	17	1,030	200
7	M	44	7	2	43	18	1,030	300
8	F	62	14	3	30	8	520	75
9	M	55	8	3	37	10	1,695	300
10	M	52	6	4	55	20	1,780	400
11	M	58	10	3	38	9	1,355	250
12	M	62	11	2	35	14	1,153	200

*Hoehn and Yahr stage was determined on range 1–5. UPDRS-III was determined with and without medication during the study. UPDRS-III is missing for the patient 3 due to interrupted measurement. The levodopa equivalent doses for medication of each patient has been calculated according to Ref. (39). The test dose was 50% higher than the patients’ normal medication dose*.

All measurements were done in the morning when the patients had been about 10–12 h without antiparkinsonian medication. The patients did neither have breakfast nor coffee before the measurement. UPDRS-III score was determined by an experienced neurologist. The rigidity and rest tremor assessments during the measurements were conducted by the measurement person.

All parts of the measurements were done while the patient was sitting upright, with their feet on the ground, on a wooden stool which had no armrests. The condition of patients was adequate when taking into account the UPDRS-III score and thus all of them were able to sit throughout the measurement. The patients were let to rest on their hospital bed between the measurement phases if needed.

### 2.2 Measurement Protocol

EMG and kinematic measurements were used to observe effects of levodopa during the levodopa challenge test. Before attaching the EMG electrodes, the surface of the skin beneath was properly cleaned with ethanol wetted cotton pads. Disposable Ag/AgCl surface electrodes (Medicotest M-00-S) were placed on top of left and right biceps brachii muscle, below the belly of the muscle with interelectrode distance 3 cm. The reference electrode was placed to an inactive point on the lateral side of brachium, 6–7 cm from the recording electrodes. The whole measurement was done without detaching the electrodes in between. For recording arm kinematics, triaxial accelerometers (MEAC-X, ±10 g Mega Electronics) were attached to anterior side of forearm, halfway between the wrist and the elbow of both arms, to record the movement of arms during the measurement. The signals were recorded with wireless ME6000 biosignal monitor (Mega Electronics Ltd., Kuopio, Finland) with sampling rate 1,000 Hz. The resolution was 1 µV for EMG acquisition and 2 milligravity for acceleration acquisition. The wireless measurement provides a shield from unwanted noise in the signals.

The measurement took place four times in total: before levodopa dose (phase I), 30 min after levodopa dose (phase II), 60 min after levodopa dose (phase III). After the levodopa challenge test was over, the patient was guided how to return into his daily medication rhythm depending on the medication response he was having. One more measurement (phase IV) was done 60 min after the patient had taken his personal medication dose.

#### 2.2.1 Task 1: Isometric Elbow Flexion

The patient was asked to hold his elbows in 90° angle with palms facing upwards. The elbows were not allowed to be supported by body sides. The patient held arms in this position for 30 s and was advised to not restrict possibly emerging tremor during the task.

#### 2.2.2 Task 2: Passive Elbow Flexion–Extension

The patient was asked to relax his arms on top of his feet. Then the patients’ elbow joint was flexed and extended periodically by holding other hand on the elbow joint and another on patients hand to allow natural track of movement. The patient was advised to not act or counteract with the movement. The measurement started after the patient relaxed his arm completely and did not perform any voluntary movements. The flexion–extension movement was repeated 9–10 times for each arm separately.

### 2.3 Analysis

The tasks were segmented from the measurement and the signals checked for artifacts and inconsistencies. The measurement of one patient was interrupted by other treatment and could not be proceeded along the protocol. The patient was omitted from the analysis.

The EMG and the kinematic signals were preprocessed for the analysis by removing possible baseline drift with smoothness priors method (40). The method resembled a high pass filter with cut off frequency 10 Hz for the EMG and 2 Hz for the kinematic signals. Then the EMG and kinematic signals were divided to short epochs of 1,024 ms with overlap 768 ms for isometric elbow flexion and 512 ms with overlap 384 ms for passive flexion–extension measurement. In the following analyses, the parameters and the histograms are first calculated for the epochs separately and then averaged over the epochs.

#### 2.3.1 Task 1: Isometric Elbow Flexion

Parameters characterizing EMG and kinematic signals were calculated for the signals measured during the isometric elbow flexion. The parameters were calculated in similarly to Ref. (11, 12). EMG shape characterizing parameters kurtosis (KURT), SD, root mean-square value (RMS), median frequency (MDF), sample entropy (SampEn), correlation dimension (D2), determinism (DET), and recurrence of bursts (REC) were determined for EMG signals. Further, parameters characterizing kinematic signals, root mean-square value (ARMS), sample entropy (ASampEn), and cross sample entropy (CSampEn), were determined. The group mean and SD over patients were calculated for each phase.

The analysis was expanded by calculating 50 bin sample histograms for the EMG signals. Then the left and right side EMG histogram were concatenated for each patient and each measurement resulting four histogram-vectors for each patient, a total of 11 × 4 vectors.

These vectors are used as the feature vectors of principal component approach. In this analysis, the directions in which the data has the greatest variance are determined.

The feature vectors *z_j_* can be modeled with linear model,

(1) $$z_{j}=H\theta_{j}+v_{j},$$

where *H* is the model matrix containing the basis vectors ϕ_1_ … ϕ*_k_* as columns. The basis vectors are the directions in which the data has the greatest variance. The parameter θ*_j_* contains the principal components. The parameter *V_j_* contains the model error. Each feature vector can be expressed as a linear combination of basis vectors multiplied by principal components

(2) $$z_{j}=\phi_{1}\;\theta_{j}(1)+\phi_{2}\;\theta_{j}(2)+\cdots +\phi_{K}\;\theta_{j}(K)+v_{j}.$$

The linear model can be presented in matrix form if the data set consists of multiple measurements or patients. In this work, feature matrix *Z* is formed from feature vectors of every subject and every measurement (11 × 4 feature vectors). Now a corresponding linear model can be written

(3) Z=Hθ+v,

where θ is the matrix of principal components and *v* the matrix of errors. The basis vectors were selected so that they are the eigenvectors of experimental correlation matrix

(4) $$R=\frac{1}{M}\Sigma_{j=1}^{M}z_{j}z_{j}^{T}=\frac{1}{M}ZZ^{T}.$$

With this selection, the first basis vector ϕ_1_ is the best mean-square fit for the data set *Z*, the vector ϕ_2_ is the best mean-square fit for the residual of the first fit and further. Four basis vectors ϕ_1_ ∙ ϕ_4_ (BV1–BV4 from this on) of largest principal components were chosen to represent the original feature vectors. The principal components can be solved from the linear model in the least-squares sense

(5) $$\theta ={\left(H^{T}H\right)}^{-1}H^{T}Z=IH^{T}Z.$$

Since the eigenvectors of *R* are orthonormal, *H^T^H* is a unit matrix.

#### 2.3.2 Task 2: Passive Elbow Flexion–Extension

The EMG signals during passive elbow flexion–extension were analyzed with time dependent spectrum approach. The epoch length was 512 ms with 386 ms overlap. Short time Fourier transform was calculated for the epochs with the spectrogram function of MATLAB (MathWorks, USA). The frequency range was set between 0 and 200 Hz, since the spectral power above 200 Hz was non-significant. The spectrum was observed visually and quantified by calculating mean power spectral density for each measurement. The values for the phases II, III, and IV were normalized with each patients phase I value to make the values comparable to other patients.

#### 2.3.3 Statistical Tests

All statistical tests were performed so that the phases of each patient were compared to the phase I. Wilcoxon signed rank test was used to determine the significance of changes in the principal components, the spectrum means and the EMG and the kinematic parameters.

## 3 Results

### 3.1 UPDRS-III

During the levodopa challenge test, UPDRS-III score of the patients changed from (37 ± 8) to (13 ± 5) indicating significant improvement of motor symptoms (Table 2). The decrease ranged from 48 to 88% in individuals and is considered a positive outcome for DBS installation. Rigidity was the most common symptom among the patients, but a majority showed also rest tremor. The Table 3 shows the group mean of the upper limb rigidity and rest tremor which were graded with UPDRS-III scale. Before the levodopa administration (phase I) the rigidity differed only slightly between the left and the right hand whereas the rest tremor seemed to be stronger on the right side. The rigidity and the rest tremor decreased already 30 min after the administration of levodopa (phase II). The effect became stronger and alleviated the rest tremor totally in the phase III, also the rigidity continued to decrease. In the last phase, the rigidity and the rest tremor began to increase indicating that the levodopa dosage given in levodopa challenge relieves the motor symptoms of the disease more than the patients’ personal medication.

**Table 2:** UPDRS-III score and limb rest tremor and rigidity during the measurement phases I–IV.

	UPDRS-III	Tremor	Rigidity
	
Range	0–108	0–16	0–16
I	37.2 ± 7.9	1.9 ± 2.4	6.9 ± 3.2
II	–	0.4 ± 0.5	3.0 ± 3.2
III	12.7 ± 5.0	0.0 ± 0.0	2.0 ± 2.5
IV	–	0.5 ± 1.2	3.5 ± 2.4

*The whole UPDRS-III was done in phases I and III, the limb rigidity and rest tremor assessment were done in each phase I–IV. The maximum of each item in UPDRS-III is 4, though the whole range of limb tremor and rigidity is 0–16*.

**Table 3:** Parameters of isometric tension task.

	I		II		III		IV	
	Left	Right	Left	Right	Left	Right	Left	Right
**UPDRS-III of upper limb**								
Rigidity	2.00 ± 1.34	1.82 ± 0.98	1.00 ± 1.34	0.64 ± 1.03**	0.45 ± 0.93**	0.27 ± 0.90**	1.27 ± 1.56*	0.82 ± 0.60*
Tremor	0.50 ± 0.67	1.08 ± 1.31	0.08 ± 0.29	0.33 ± 0.65	0.00 ± 0.00	0.00 ± 0.00	0.25 ± 0.45	0.25 ± 0.62

**EMG-parameters**								
KURT	4.79 ± 1.66	4.33 ± 0.65	4.42 ± 0.60	4.20 ± 0.66	4.49 ± 0.71	4.18 ± 0.55	4.34 ± 0.68	4.14 ± 0.49
DEV	0.34 ± 0.03	0.35 ± 0.03	0.34 ± 0.02	0.35 ± 0.02	0.34 ± 0.02	0.35 ± 0.03	0.35 ± 0.03	0.36 ± 0.02
RMS	46.8 ± 27.8	36.9 ± 10.1	65.1 ± 55.9	69.2 ± 75.7	67.7 ± 34.0	70.2 ± 58.5	49.4 ± 20.0	64.0 ± 37.9
MDF	71.0 ± 18.5	66.2 ± 14.8	67.3 ± 11.0	67.4 ± 15.3	66.0 ± 12.7	68.4 ± 13.5	69.0 ± 16.8	67.6 ± 16.9
REC	8.2 ± 3.5	8.9 ± 5.3	7.5 ± 2.9	9.0 ± 7.2	8.0 ± 3.0	6.9 ± 2.8	8.0 ± 2.9	8.6 ± 5.3
DET	9.7 ± 6.2	15.3 ± 15.6	9.7 ± 4.2	15.3 ± 15.8	11.9 ± 9.1	10.6 ± 9.4	10.5 ± 5.1	14.7 ± 14.1
SampEn	1.11 ± 0.26	1.16 ± 0.24	1.15 ± 0.13	1.17 ± 0.26	1.11 ± 0.22	1.20 ± 0.20	1.15 ± 0.19	1.18 ± 0.21
D2	6.23 ± 0.83	6.21 ± 1.03	6.40 ± 0.48	6.23 ± 1.30	6.26 ± 0.64	6.55 ± 0.69	6.26 ± 0.53	6.28 ± 1.11

**Kinematic parameters**								
ARMS	0.06 ± 0.02	0.08 ± 0.09	0.06 ± 0.03	0.09 ± 0.11	0.08 ± 0.05	0.06 ± 0.04	0.09 ± 0.08	0.10 ± 0.08
ASampEn	1.14 ± 0.28	1.00 ± 0.37	1.11 ± 0.30	1.02 ± 0.36	1.11 ± 0.44	1.10 ± 0.26	0.97 ± 0.47	0.86 ± 0.37
CSampEn	1.28 ± 0.21	1.20 ± 0.32	1.25 ± 0.21	1.22 ± 0.26	1.26 ± 0.31	1.28 ± 0.14	1.23 ± 0.24	1.16 ± 0.20

**PCA of EMG**								
PC1	0.50 ± 0.26		0.29 ± 0.20		0.22 ± 0.13*		0.32 ± 0.22**	
PC2	0.65 ± 0.30		0.85 ± 0.15		0.91 ± 0.08*		0.80 ± 0.19**	
PC3	0.39 ± 0.28		0.32 ± 0.18		0.25 ± 0.21		0.35 ± 0.33	
PC4	0.62 ± 0.27		0.62 ± 0.15		0.58 ± 0.11		0.62 ± 0.14	

UPDRS-III score for arm rigidity and rest tremor decreased in the phases I–III and increased in the phase IV compared to phase III. There were only slight changes in EMG and kinematic parameters between the phases, none of which was significant. The principal component approach showed significant difference between the phases I and III and between the phases I and IV. Values presented in format (mean ± SD). Significant change to first measurement *p < 0.05, **p < 0.01.

### 3.2 Task 1: Isometric Elbow Flexion

The EMG and the kinematic signals for a single patient are shown in Figure S1 in Supplementary Material. There were some differences in the EMG signals between the different phases. In the phase I, the left hand EMG contained bursts which decreased in the phases II–IV. On the right hand side, the signal amplitude increased from the phase I to the phase III. The kinematic signals showed slightly less changes between the phases I–IV. The amplitude was greatest in the phase II, but the frequency was slightly high (around 9 Hz) compared to typical Parkinsonian rest tremor (4–6 Hz).

The calculated EMG and kinematic parameters (Table 3) differed slightly between the phases. However, the deviation was high and there were no statistically significant changes either in the EMG or the kinematic parameters. According to the UPDRS-III score, majority of the patients suffered from rest tremor. Traditionally this is easily picked up by kinematic measurement. However, in this study, the kinematic measurement and the signal RMS values showed that rest tremor is generally very low.

The characterization of the EMG signals was taken further by including principal component analysis. The basis vectors for characterization of the histograms are presented in Figure S2 in Supplementary Material. The first and the second BV characterized the histogram height and width, which are closely related to the EMG signal characteristics in PD. The third BV characterized the side differences in histogram peak height, whereas the fourth BV was a mixture of peak width and side differences. The principal components which showed the greatest difference between the phases, PC1 and PC2, are shown in Figure S3 in Supplementary Material. These two principal components are related to the signal burstiness. It is seen that in the phase II there are varying responses between the patients. While the PC1 decreases and the PC2 increases for most patients, opposite paths are observed also. In the phase III, the effect of medication is more homogeneous, nearly all of the patients experience decrease in the PC1 and increase in the PC2. In the phase IV, the response is similar to the phase III, but milder. Means and SDs of the coefficients PC1–PC4 are shown in Table 3. For the PC1 and the PC2, it indicates the same results than the Figure S2 in Supplementary Material. It is seen that the PC3 changes similarly than the PC1, indicating that there is a decrease in side difference, but not significant. There was practically no change in the PC4 between the phases.

### 3.3 Task 2: Passive Extension–Flexion Task

The time dependent spectrum of EMG activation during the passive flexion–extension task showed muscle activity even though the patients were not voluntarily tensing their muscles. The involuntary muscle activity was strongest in the phase I, while a decreasing trend was observed toward phases II and III. In the phase IV, a slight increase in activity compared to phase III is observed. Muscle activation in phases I and III for both hands of each patient is shown in Figure S4 in Supplementary Material. The decrease in involuntary activity is clear in most of the patients. We hypothesize that this is an indication of Parkinsonian rigidity. The EMG amplitudes are not directly comparable between the patients. However, the amplitudes can be compared between the measurement phases of one patient, since all the phases were measured in the same session without moving or detaching the electrodes in between. The normalized mean power spectral density decreased to (0.88 ± 0.40, 0.63 ± 0.31**) (left, right) in the phase II (not shown in the figure), to (0.62 ± 0.39*, 0.51 ± 0.21**) in the phase III and increased again to (0.65 ± 0.40*, 0.68 ± 0.28**) in the phase IV (not shown in the figure). The change was significant compared to the phase I in the phases III and IV on the left arm, and in the phases II-IV on the right arm. Significances **p < 0.01, *p < 0.05.

## 4 Discussion

In this study, 12 patients with advanced PD went through tests to determine their applicability for DBS treatment. During the levodopa challenge test, the muscle activity and arm movements of the patients were measured, and 11 of them were analyzed. The results of the study proved that a wireless and wearable device combined with the presented analysis can be used to objectively monitor the muscle activity during levodopa challenge test.

The main finding of the study is that levodopa challenge test changes the characteristics of EMG and kinematic signals in patients with advanced PD. The proposed principal component approach suggests that the morphology of EMG changes due to levodopa administration so that the EMG histogram peak lowers and widens. The clinical indication of this is the alleviation of PD symptoms. Variation in the phase II suggests that the begin of medication response varies between the patients. The third PC shows slight decline in side difference, but the change was non significant. PD typically begins unilaterally and these results suggest that even though medication relieves the symptoms bilaterally, it failed to lessen the side difference between the left and the right arm. The nearly absent change in PC4 indicates that most of the differences in histograms are described already by the three first PC’s and it is only used to fine tuning the histogram shape. The strength of the principal component approach is in the core of the method. It relies on determining the directions of data variation in the data set and thus is tailored to find the differences in that particular data set. This is a slight shortcoming of the method at the same time. The method needs a training set and it cannot be used for a single measurement.

The second main finding is that the effect of levodopa can be seen also in passive flexion–extension task. It was found that levodopa dosage decreases involuntary muscle tone in patients of PD. The results follow trend that is similar to the isometric task. In the phase II, there is more variation in the results, but in the phase III there is a clear decrease in involuntary muscle activation. In the phase IV, it is slightly increased compared to III, but still closer to phase III than phase I. Similar results for measurement during DBS treatment has been observed by Levin et al. (30). When comparing the UPDRS-III limb rigidity, similar trend is observed. We hypothesize that the decrease in rigidity is a result from decreased involuntary muscle activity. Thus, the passive flexion–extension measurement is connected to the Parkinsonian rigidity, and can be used to measure it. We are aware of the difficulties which this method poses: (1) the rate of limb flexion–extension was not controlled precisely and (2) the patients’ voluntary movement cannot be perfectly ruled out. The (1) can affect to power spectral density, but we assume that the effect is not significant since the measurement person used same speed for each patient (slightly less than 1/s). Also it can be speculated, that while rigidity could decrease the rate of movement, which would also decrease the difference between phase I and phase III. The (2) can cause false (voluntary) movements during passive flexion–extension cycle. However, the patients were advised to keep their hand in rest while the movement and the measurement was not began before the measurement person felt the patient was not voluntarily contracting their muscles. While it can be argued that patients learn to relax their hand throughout the measurement, this is not the case according to the data: during the fourth phase, most of the patients experienced increased rigidity which is also picked up by EMG measurement.

The third main finding was that unlike in earlier studies, the Parkinsonian symptoms were not visible in the parameters calculated from EMG and kinematic signals during the isometric task. Even patients who presented rest tremor during the UPDRS-III assessment, did not show significant tremor in the kinematic signals. This is an atypical finding since rest tremor is easily picked up by kinematic sensors. Multiple factors can affect to this. It is possible that patients (despite the advice) were restricting their tremor during the isometric measurement. This is quite common along the patients in general. The tremor in PD is mainly rest tremor which disappears during posture or kinetic tasks. It is possible that the isometric tension measurement measures postural tremor and, therefore, is not compatible method to measure rest tremor. However, contrary results have been observed in earlier studies (12, 38). In the third and fourth measurement, rest tremor is absent due to the medication. The patients in this study were going through a series of clinical trials which tell us if the patients would benefit from DBS treatment. Patients older than 70 years may tolerate DBS less well than younger patients like in this study. This affects to our patient selection, and it could be possible that previously mentioned issues are emphasized compared to general population of PD patients. This notion is backed up with the fact that the EMG signals show similar values for parameters for healthy controls as in our earlier study, even when the patients were off-medication. However, principal component approach is more capable to extract information from the EMG signals. In this approach, we see clear changes between the signals during the medication dosage. However, the number of subjects is small for drawing definitive statistical conclusions.

The results of the study indicate that the patients’ response to levodopa during levodopa challenge is stronger than the response to their own personal medication. This was an expected result: since the levodopa dosage in levodopa challenge test is 1.5 times the patients’ optimal dose, the response is also pronounced. However, this does not imply that the patients’ medication dosage is not optimal. When determining suitable medication dose, also the adverse effects have to be taken into account. During this measurement, part of the patients experienced levodopa-induced dyskinesias and some of them were struggling to keep steady during the end of the phase III measurement due to strong medication response.

The strengths of this analysis are the feasible measurements, only the wireless measurement device is needed, as comparison to other methods which typically incorporate special equipment such as manipulators to carry out the measurement. Even though the measurements are currently done with a wireless measurement device which is the size of a scientific calculator, the technology today allows this method to be directly used on even smaller devices. The analysis methods are not computer intensive which enables their use in simpler devices, for example the measurement device. Present method appears to objectively assess the effect of levodopa challenge test on muscle activity and activation patterns in advanced PD. However, there is no restriction for using the method to follow the effects of levodopa during the course of disease. With further research, this method can possibly be used also for analysis of long-time registration of medication response in PD.

## Ethics Statement

This study was approved by the Research Ethics Committee of the Northern Savo Hospital District. All subjects gave a written informed consent in accordance with the Declaration of Helsinki before the measurements.

## Author Contributions

VR: patient measurements, data analysis, and manuscript writing. EP: management of patient measurements and manuscript writing. OA: study planning and management, funding, and manuscript writing. MK: study planning and manuscript writing. PK: study planning and management, funding, and manuscript writing. SR: study planning, data analysis, and manuscript writing.

## Conflict of Interest Statement

The authors declare that the research was conducted in the absence of any commercial or financial relationships that could be construed as a potential conflict of interest.

## Supplementary Material

Figure S1 Three second segment of EMG and kinematic signals during isometric tension of left and right arm in one patient. EMG bursts decrease in phases I–III on left side, whereas EMG amplitude increases in phases I–IV on right side. There is more tremor-like activity in kinematic signals of phase II than others.

Figure S2 Basis vectors BV1–BV4 of the data set determined by PCA. BV1 denotes EMG histogram peak height, BV2 peak width, BV3 the side differences, and BV4 is a partial mixture of side differences and peak height.

Figure S3 Principal components PC1 and PC2 in phases II–IV, normalized to the phase I. The phase II and the phase III show similar features. The phase IV indicates that the effect of the patients own medication is milder than that of the levodopa test dose (phase III). The phase I for each patient is marked with a solid circle while the hollow circles indicate the change from the phase I.

Figure S4 Left and right arm EMG spectral power during passive extension–flexion task in phases I and III for each patient. White colour denotes higher spectral intensity. The EMG spectral power decreases from the phase I to the phase III in passive extension–flexion task.
