# Supplementary figures and images for: Levodopa-Induced Changes in Electromyographic Patterns in Patients with Advanced Parkinson’s Disease
Source: Front Neurol. 2018 Feb 5;9:35. doi: 10.3389/fneur.2018.00035 (PMC5807331; doi:10.3389/fneur.2018.00035)

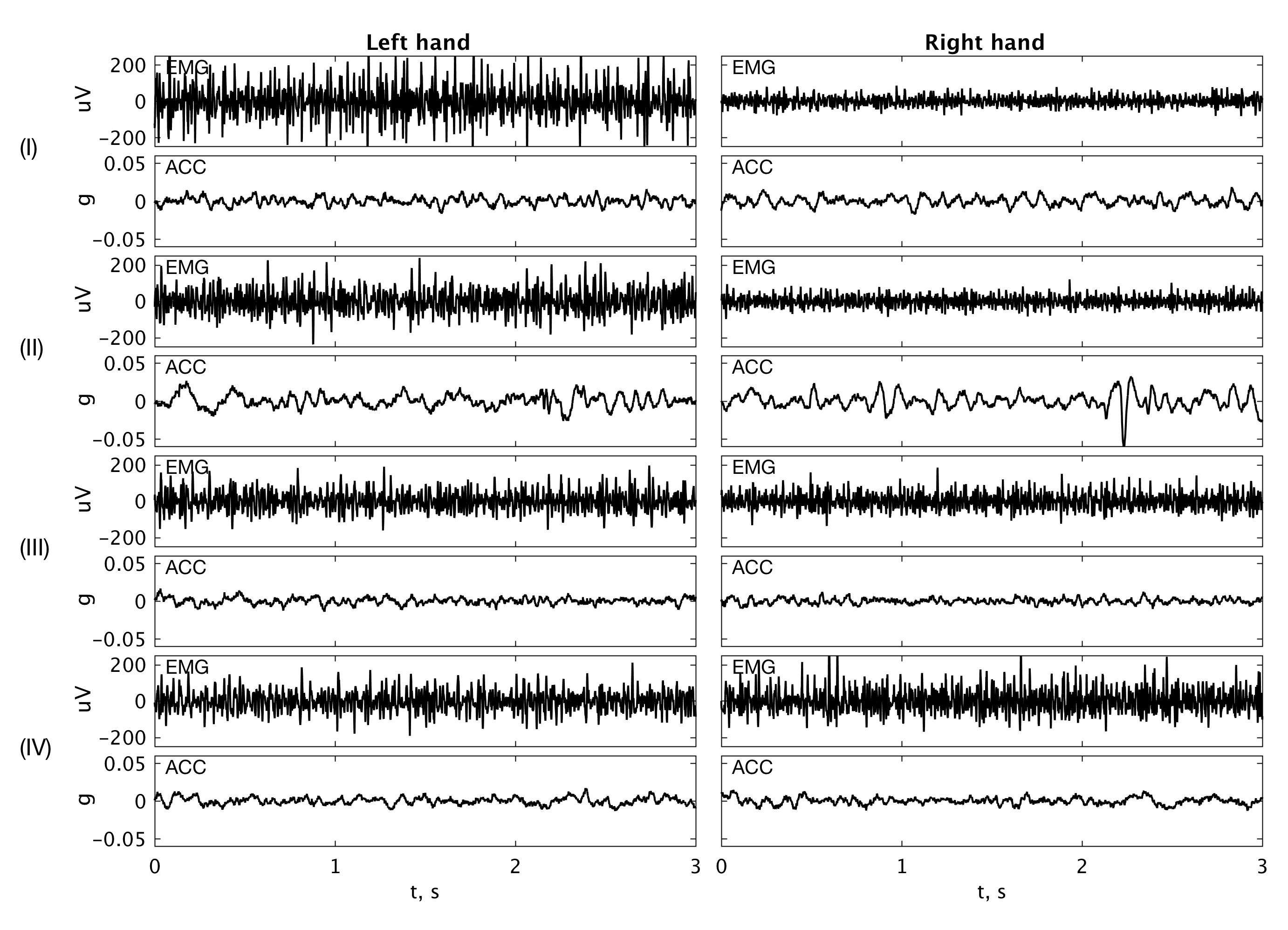

Supplement: Figure S1 — Three second segment of EMG and kinematic signals during isometric tension of left and right arm in one patient. EMG bursts decrease in phases I–III on left side, whereas EMG amplitude increases in phases I–IV on right side. There is more tremor-like activity in kinematic signals of phase II than others. [file image_1.png]

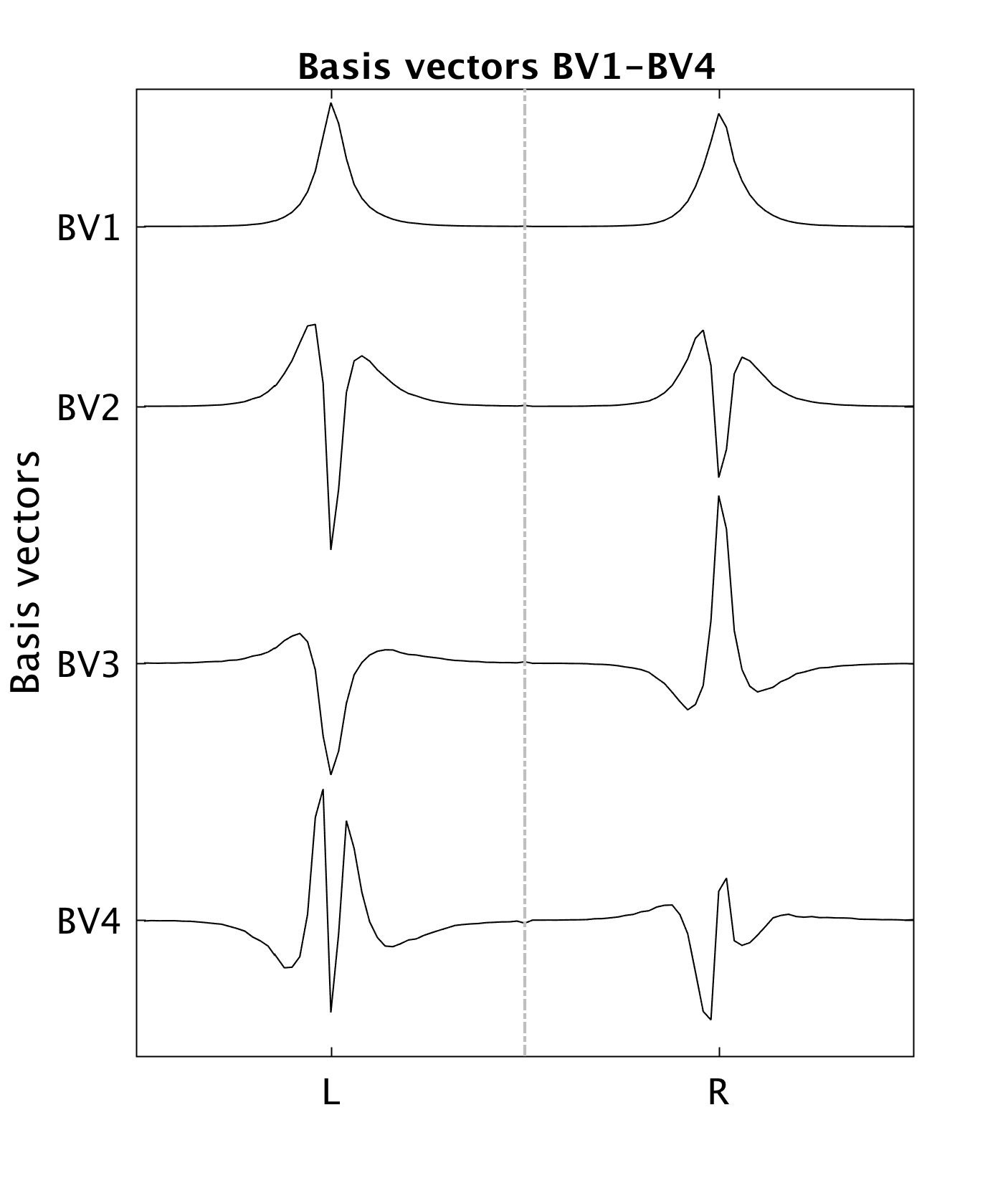

Supplement: Figure S2 — Basis vectors BV1–BV4 of the data set determined by PCA. BV1 denotes EMG histogram peak height, BV2 peak width, BV3 the side differences, and BV4 is a partial mixture of side differences and peak height. [file image_2.png]

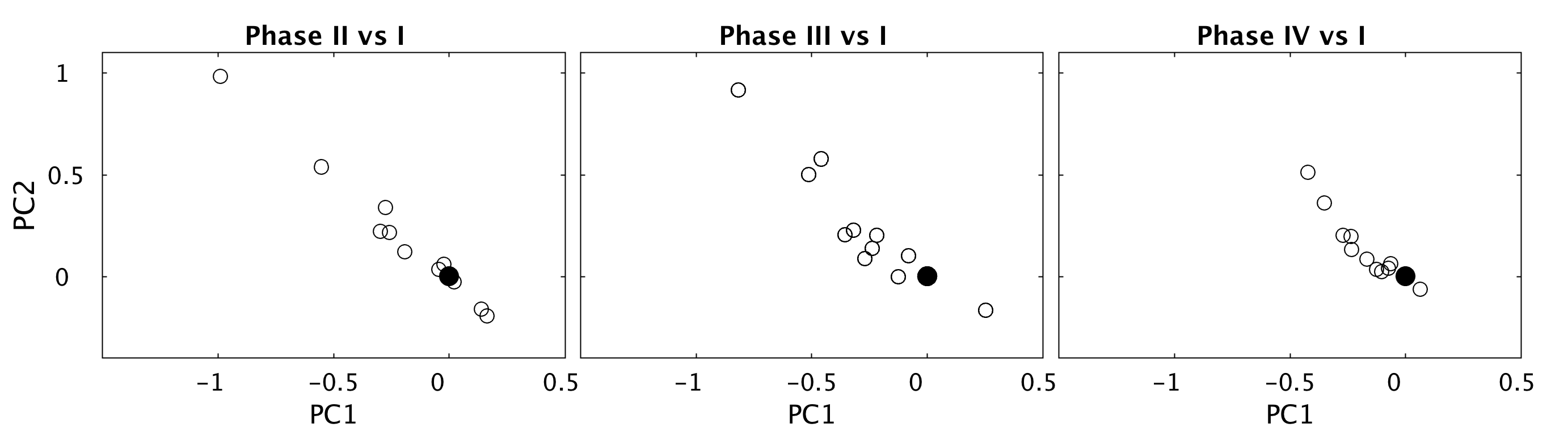

Supplement: Figure S3 — Principal components PC1 and PC2 in phases II–IV, normalized to the phase I. The phase II and the phase III show similar features. The phase IV indicates that the effect of the patients own medication is milder than that of the levodopa test dose (phase III). The phase I for each patient is marked with a solid circle while the hollow circles indicate the change from the phase I. [file image_3.png]

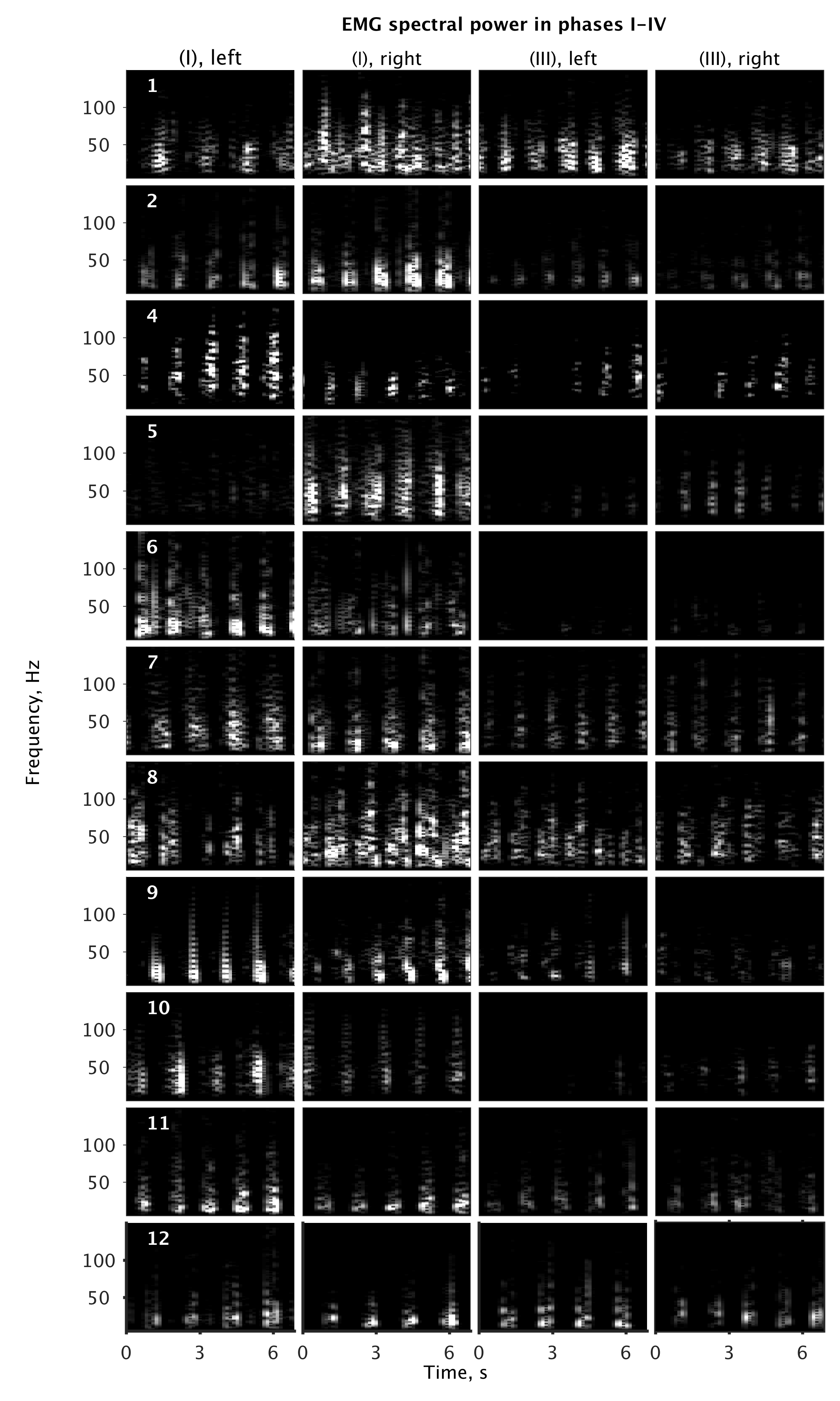

Supplement: Figure S4 — Left and right arm EMG spectral power during passive extension–flexion task in phases I and III for each patient. White colour denotes higher spectral intensity. The EMG spectral power decreases from the phase I to the phase III in passive extension–flexion task. [file image_4.png]
